# Comparison of the efficacy of different treatments after non-curative endoscopic resection of superficial esophageal carcinoma: a meta-analysis

**DOI:** 10.3389/fonc.2025.1709350

**Published:** 2025-12-18

**Authors:** Rui Wang, Li-Jun Peng, Shu-Ni Tian

**Affiliations:** 1 School of Clinical Medicine, Shandong Second Medical University, Weifang, Shandong, China; 2 Department of Gastroenterology, Qingdao Hospital of Peking University People’s Hospital, Qingdao, Shandong, China; 3 Department of Gastroenterology, Linyi People’s Hospital, Linyi, Shandong, China

**Keywords:** esophageal carcinoma, meta-analysis, non-curative endoscopic resection, radiotherapy, surgery

## Abstract

**Objective:**

This study aims to systematically evaluate the efficacy of different treatments after non-curative endoscopic resection for superficial esophageal carcinoma.

**Methods:**

Databases including PubMed, the Cochrane Library, Embase, China National Knowledge Infrastructure (CNKI), and Wanfang Data were searched from inception to November 25, 2025. The meta-analysis was performed using Review Manager version 5.3, with Stata version 15 employed for supplementary statistical assessments.

**Results:**

A total of 13 studies involving 1,304 patients were included. The results of the meta-analysis showed that the recurrence rate after non-curative endoscopic resection of superficial esophageal carcinoma was significantly higher in the observation group than in the adjuvant treatment group (OR = 3.25, 95% CI: 1.88–5.62, *P* < 0.0001). However, there was no significant difference in recurrence rate between the chemoradiotherapy group and the surgery group (OR = 0.65, 95% CI: 0.23–1.84, *P* = 0.42). Patients with lymphovascular invasion had a higher recurrence rate, which was statistically significant (OR = 4.01, 95% CI: 1.48–10.84, *P* = 0.006).

**Conclusion:**

Lymphovascular invasion is a risk factor for recurrence in patients. The adjuvant treatment group shows significantly reduced recurrence rates compared to the observation group, with no significant difference in recurrence rates between surgical and chemoradiotherapeutic approaches.

**Systematic review registration:**

https://www.crd.york.ac.uk/prospero/, identifier CRD420251108299.

## 1 Introduction

Esophageal cancer is one of the most common malignancies, ranking 11th in global incidence and seventh in mortality worldwide (1), and it is the fifth leading cause of cancer mortality in China (2), according to the Global Cancer Statistics 2022.

Superficial esophageal carcinoma is defined as carcinoma confined to the mucosa (T1a) or submucosa (T1b), irrespective of lymph node or distant metastasis. Based on the depth of invasion, mucosal esophageal cancer may be limited to the epithelium (M1), lamina propria (M2), and muscularis mucosae (M3), whereas submucosal carcinoma is classified by dividing the submucosa into thirds: SM1 (upper third), SM2 (middle third), and SM3 (lower third). A supplementary criterion specifies that in endoscopically resected specimens, where the total submucosal thickness is unknown, invasion ≤200 μm from the muscularis mucosae is classified as SM1, while invasion >200 μm is classified as SM2 (3).

Historically, esophagectomy with lymph node dissection has been the standard of care for high-risk superficial esophageal cancer (stage T1b). However, this procedure is highly invasive and carries significant risks of adverse events and mortality (4, 5). In contrast, endoscopic resection (ER), including techniques like endoscopic mucosal resection (EMR) and endoscopic submucosal dissection (ESD), offers advantages such as lower costs, shorter procedure times, fewer postoperative complications, and faster recovery. Consequently, ER has emerged as a safer alternative and is now the recommended first-line treatment for superficial esophageal cancer (6, 7).

According to the Chinese Guidelines for Screening and Early Diagnosis and Treatment of Esophageal Carcinoma (2022 Beijing), absolute indications for endoscopic resection of superficial esophageal cancer are M1 and M2 cancers, while relative indications include M3 and SM1 (8). The Japanese Esophageal Society (JES) recommends that patients with M3 invasion after ESD be managed with either close observation or adjuvant therapy (surgery or chemoradiotherapy (CRT)). For those with T1b submucosal invasion, surgery or CRT is recommended, following an assessment of the patient’s tolerance. These recommendations stem from the fact that for lesions invading to the depth of M3 or T1b, ER alone is often non-curative due to a substantially increased risk of lymph node metastasis. Consequently, the choice of subsequent management should be individualized based on the specific pathological characteristics of the lesion and the patient’s comorbidities.

Currently, there is no standardized adjuvant management following non-curative ER for superficial esophageal cancer. This meta-analysis aims to evaluate the comparative efficacy and safety of different management strategies (surgery, CRT, or observation) after non-curative ER of superficial esophageal carcinoma.

## 2 Methods

### 2.1 Protocol and registration

The inclusion criteria and methods of the analysis were pre-specified in a protocol and registered on PROSPERO (registration number CRD420251108299).

### 2.2 Literature search strategy

A systematic literature search was conducted in PubMed, the Cochrane Library, Embase, China National Knowledge Infrastructure (CNKI) and Wanfang Data, with no language restrictions, all from inception to 25 November 2025. The search employed a combination of subject headings and free-text terms. The search keywords included esophageal cancer, non-curative, chemotherapy, radiotherapy, surgery, observation, and chemoradiotherapy.

### 2.3 Inclusion and exclusion criteria

Studies were included if they involved patients who underwent non-curative ER for superficial esophageal cancer and compared different subsequent management strategies (surgery, CRT, or observation). In studies reporting both non-curative and curative ER, only data from the non-curative groups were extracted. Additionally, reviews, meta-analyses, case reports, conferences, letters, guidelines, expert consensus, theses, duplicates, and studies with unavailable full text were excluded. Studies with five or fewer patients receiving a specific adjuvant therapy or those with insufficient or ambiguous data were also excluded.

Curative resection was defined as stage T1a-M1 or T1a-M2 with negative lateral and basal margins (R0 resection) and the absence of lymphovascular invasion (9). In contrast, non-curative resection was characterized by any of the following criteria: microscopically positive margins (R1 resection), the presence of lymphatic or vascular invasion, or a lesion depth reaching T1a-M3 or T1b.

### 2.4 Data extraction and quality assessment

Two investigators independently screened the literature against the inclusion criteria, cross-checked their work, and resolved any disagreements through discussion or by consulting a third researcher. The following information was extracted from each study: author, publication year, post-ER treatment strategy, number of patients, and recurrences events. The primary outcome was the number of recurrences. The methodological quality was evaluated using the Newcastle–Ottawa Scale (NOS) (10) for observational studies. Two investigators independently assessed the quality of the included studies and cross-checked their assessments. In the event of a dispute, the opinion of a third researcher was sought to reach a consensus.

### 2.5 Statistical analysis

Data analysis was performed using Review Manager version 5.3, with Stata version 15 employed for supplementary statistical assessments. Heterogeneity was assessed by examining the *P*-value from Cochran’s Q test and the *I*² statistic. When *P >*0.10 and/or *I*^2^ <50%, indicating low heterogeneity, a fixed-effects model was used. Conversely, when *P <*0.10 and *I*^2^ >50%, suggesting significant heterogeneity, a random-effects model was employed. Results were expressed as odds ratios (OR) with 95% confidence intervals (CI). Sensitivity analysis was conducted using a stepwise elimination approach. Publication bias was assessed using Egger’s test at a significance level of *α* = 0.05.

## 3 Results

### 3.1 Literature search and study selection

**Figure 1:**
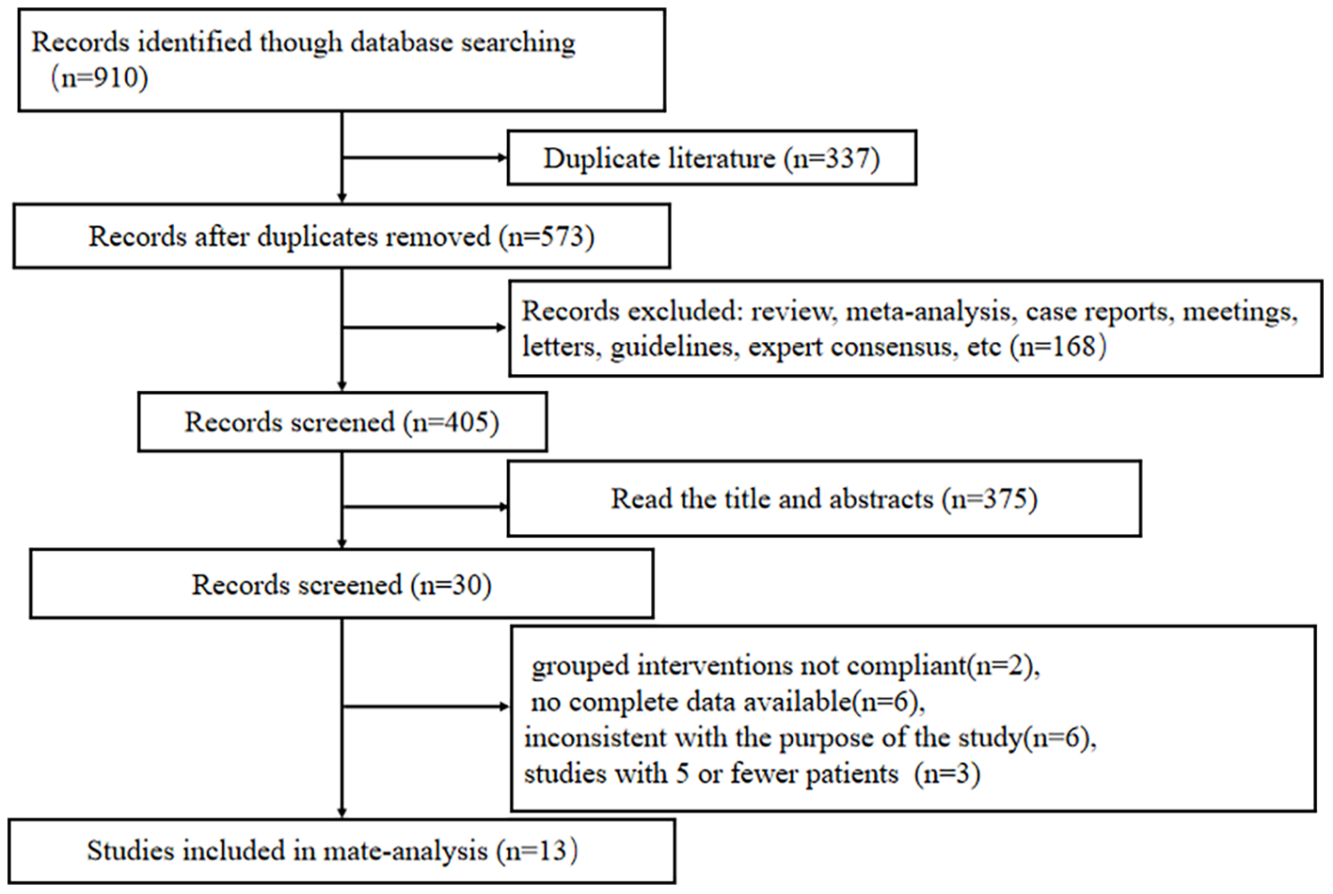
Screening process for the inclusion of literature.

The initial search identified 910 records. After removing duplicates, reviews, meta-analyses, case reports, and other irrelevant publications, we reviewed the titles and abstracts of 405 articles and evaluated 30 potentially eligible studies for inclusion. Following full-text assessment, 17 studies were excluded due to insufficient or ambiguous data or because adjuvant therapy was offered to five or fewer patients. The study selection process is described in **Figure 1**. The characteristics of the included studies are shown in **Table 1**. All included studies were retrospective, with squamous cell carcinoma accounting for the majority. The results of the quality assessment are shown in **Table 2**. The NOS has eight items, with a maximum score of 9 points. A score ≤4 points indicates low quality, 5 to 6 points indicate medium-quality, and ≥7 points indicate high quality. The median score of the included studies in this meta-analysis was 8 points, indicating that all were of moderate to high quality.

**Table 1:** Characteristics of the included studies.

Study	Pathological type	Treatment arms	*n*	Recurrence, *n*	Accessed outcomes
Tomohiro Kadota 2022 (11)	ESCC	ER + S ER + CRT ER+O	18 50 21	2 2 5	OS, RFS, CRR
Yasufumi Koterazawa 2018 (12)	ESCC	ESD + S ESD + CRT	28 31	0 5	OS, DSS, AE
Byeong Geun Song 2021 (13)	ESCC	ESD + S ESD + O	29 23	5 3	OS, RFS, DSS
Gen Suzuki 2022 (14)	ESCC	ESD + CRT ESD + S	34 26	10 5	OS, DFS
Tanaka T 2019 (15)	ESCC	ER + S ER + CRT	19 33	0 6	OS, RFS
Hongna Lu 2024 (16)	ESCC	ER + RT ER + O	9 11	0 4	PFS
Emi M 2022 (17)	ESCC	ER + S ER + CRT	30 37	2 3	OS, DSS
Kanie Y 2021 (18)	ESCC	ER + S ER + CRT	56 52	0 2	OS, DSS
Xu Yang 2023 (19)	ESCC	ER + RT ER + O	47 114	2 20	OS, DFS
Sakiko Naito 2021 (20)	ESCC	ER + CRT ER + S ER + O	33 29 163	2 0 7	OS, DSS, RFS
Osamu Hisano 2018 (21)	ESCC	ER + RT ER + O	13 14	0 4	AE, OS, DSS
Mengxue Chen 2020 (22)	Not mentioned	ER + S/CRT ER + O	25/12 27	7 11	OS
Yoshinobu 2025 (23)	ESCC	ER + S ER + CRT	160 160	15 28	OS, RFS

ESCC, esophageal squamous cell carcinoma; S, surgery; CRT, chemoradiotherapy; O, observation; RT, radiotherapy; OS, overall survival; RFS, recurrence-free survival; CRR, cumulative relapse rate; DSS, disease-specific survival; AE, adverse event; DFS, disease-free survival; PFS, progression-free survival.

**Table 2:** Risk-of-bias evaluation of the included studies.

Study	Representativeness of the exposed cohort	Selection of the non-exposed cohort	Ascertainment of exposure	Demonstration that outcome of interest was not present at start of study	Comparability	Assessment of outcome	Adequacy of follow up	Completeness of observation	Total score
Tomohiro Kadota 2022 (11)	1	1	1	1	0	1	0	1	6
Yasufumi Koterazawa 2018 (12)	1	1	1	1	2	1	0	1	8
Byeong Geun Song 2021 (13)	1	1	1	1	0	1	0	1	6
Gen Suzuki 2022 (14)	1	1	1	1	1	1	0	1	7
Tanaka T 2019 (15)	1	1	1	1	0	1	1	1	7
Hongna Lu 2024 (16)	1	1	1	1	2	1	0	1	8
Emi M 2022 (17)	1	1	1	1	1	1	0	1	8
Kanie Y 2021 (18)	1	1	1	1	0	1	1	1	7
Xu Yang 2023 (19)	1	1	1	1	2	1	0	1	8
Sakiko Naito 2021 (20)	1	1	1	1	2	1	1	1	9
Osamu Hisano 2018 (21)	1	1	1	1	2	1	1	1	9
Mengxue Chen 2020 (22)	1	1	1	1	Not mentioned	1	0	1	6
Yoshinobu 2025 (23)	1	1	1	1	2	1	1	1	9

### 3.2 Meta-analysis results

#### 3.2.1 Recurrence according to management strategy

**Figure 2:**
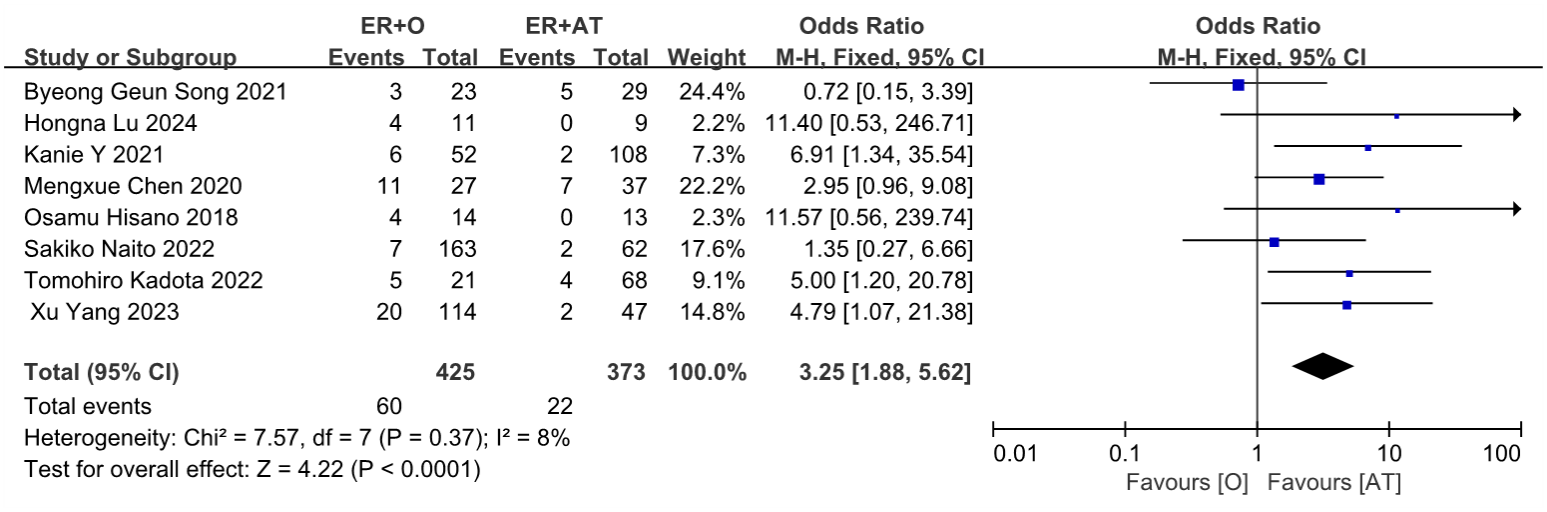
Forest plot comparing the recurrence between ER followed by the observation and ER followed by adjuvant treatment.

Eight studies reported data on recurrence rates after comparing observation versus adjuvant therapy after non-curative ER for superficial esophageal cancer. There were 60 recurrences in the observation group (*n* = 425) and 22 recurrences in the adjuvant treatment group (*n* = 373). Given the absence of significant heterogeneity, a fixed-effects model was used for analysis. The meta-analysis results demonstrated a statistically significant difference in recurrence rates: the recurrence rate in the observation group was higher than that in the adjuvant treatment group (OR = 3.25, 95% CI: 1.88–5.62, *P* < 0.0001) (**Figure 2**).

**Figure 3:**
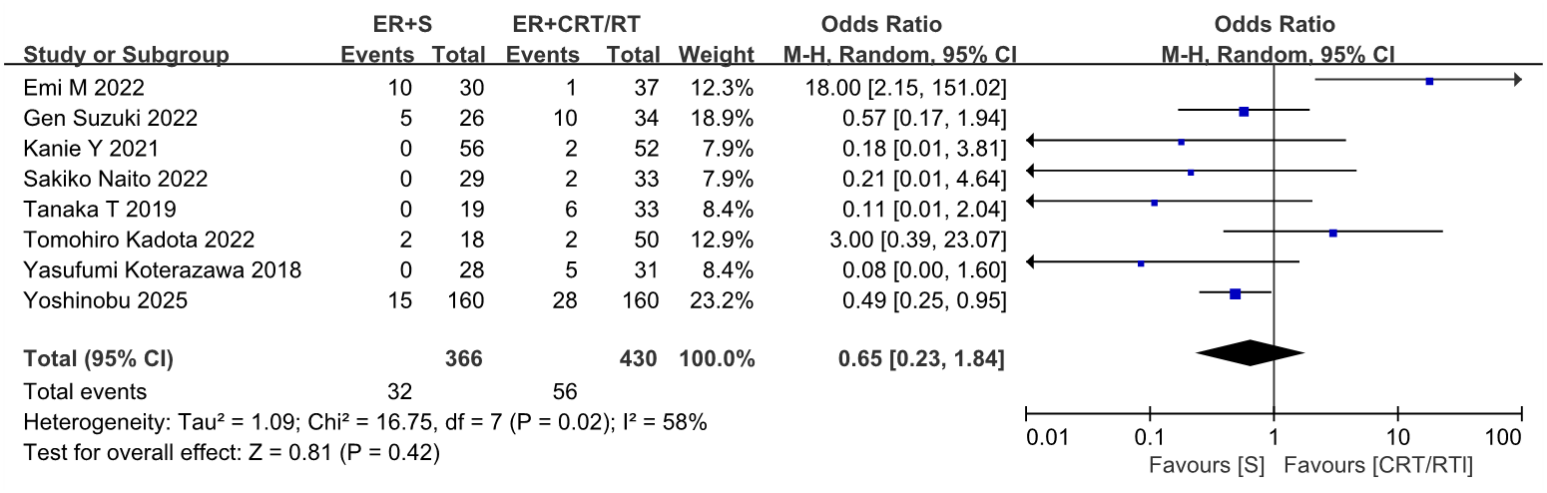
Forest plot comparing the recurrence between ER followed by surgery and ER followed by CRT/RT.

Eight studies compared the recurrence rates between adjuvant CRT/RT and surgery after non-curative ER. These studies included 430 patients in the CRT group and 366 in the surgery group. Due to moderate heterogeneity, a random-effects model was used for analysis. The results showed no statistical difference in recurrence rates between the two groups (OR = 0.65, 95% CI: 0.23–1.84, *P* = 0.42) (**Figure 3**).

#### 3.2.2 Risk factors for recurrence

**Figure 4:**
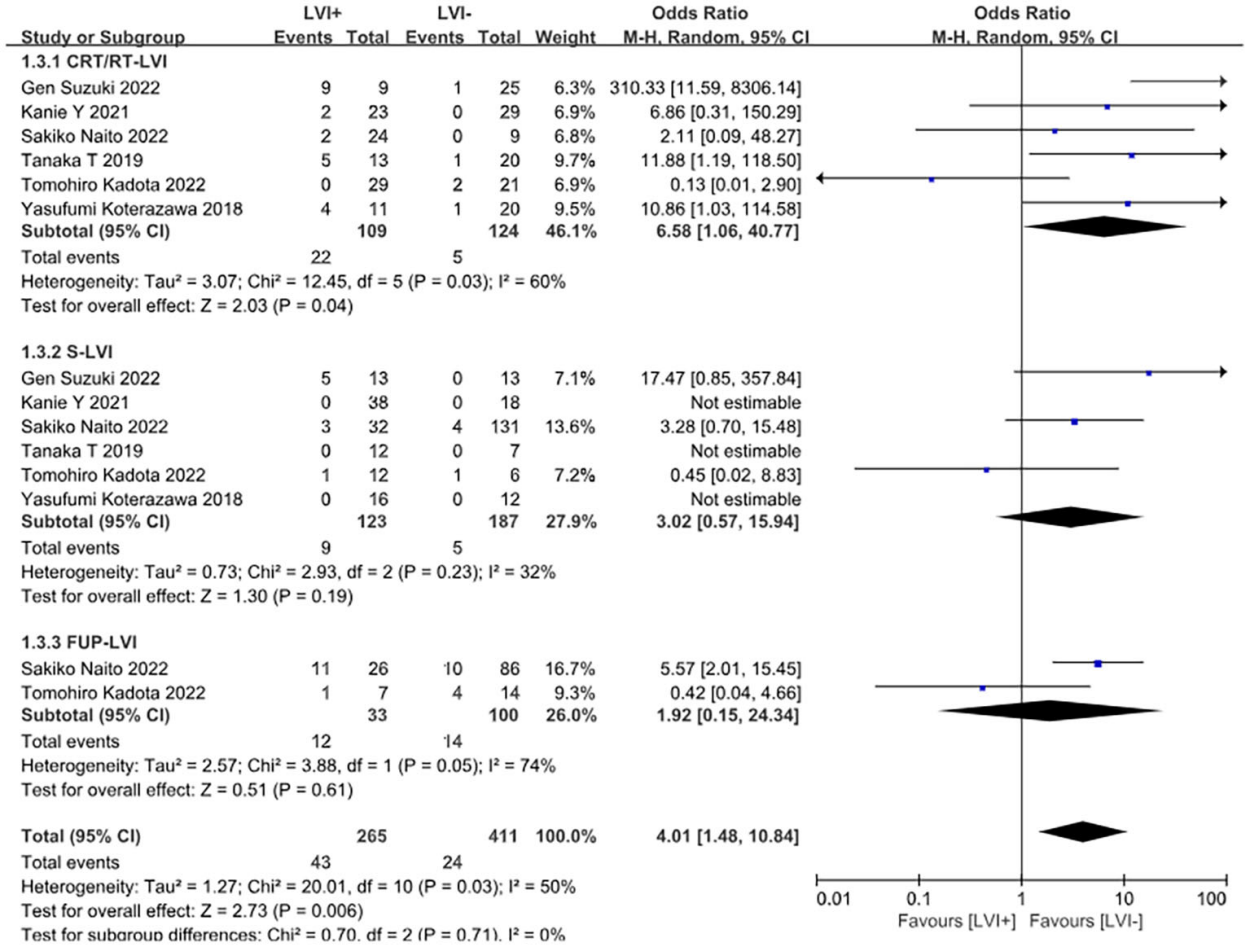
Forest plot comparing the recurrence of different interventions (CRT/RT, surgery, and observation alone) after endoscopic treatment in patients with positive versus negative lymphovascular infiltration.

**Figure 5:**
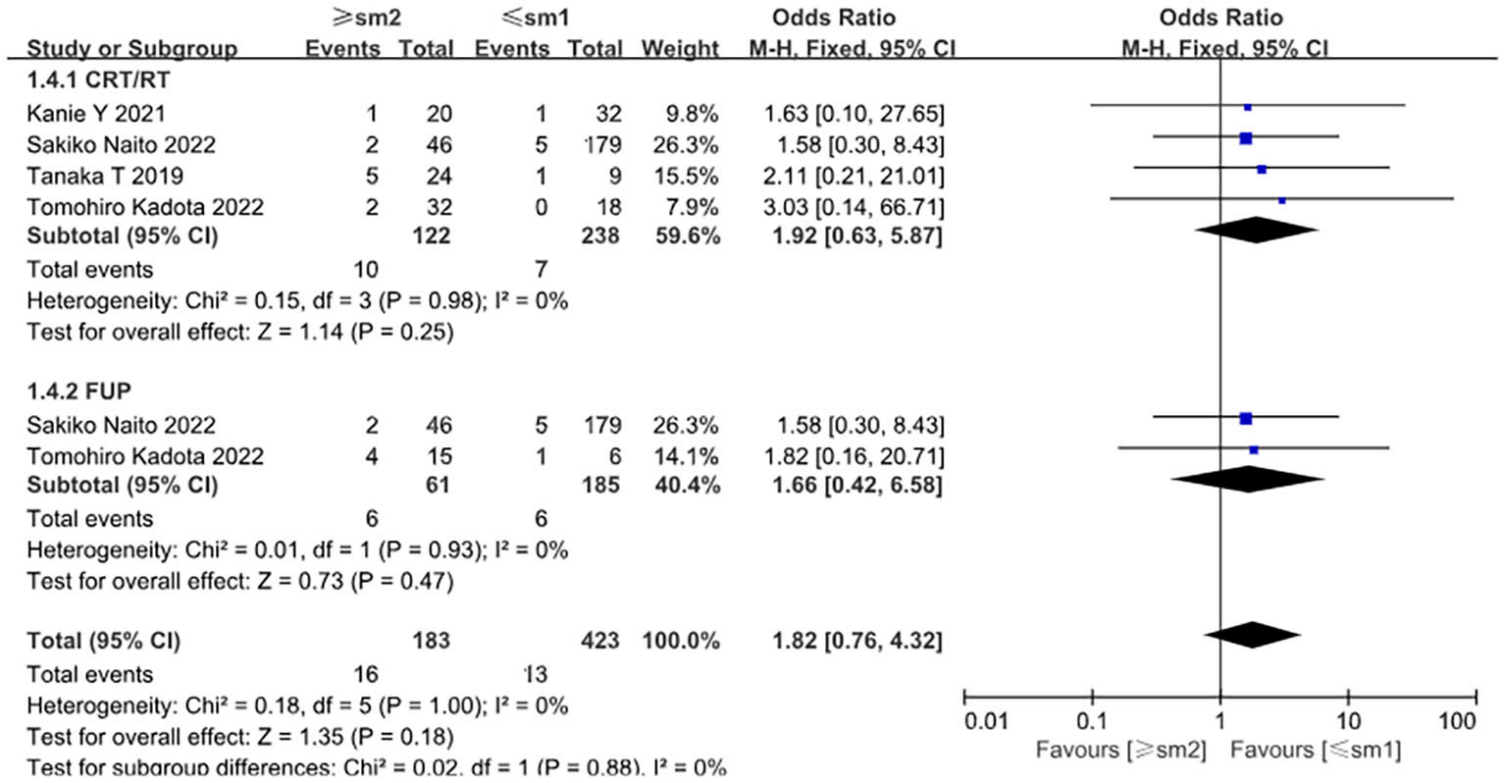
Forest plot comparing the recurrence of different interventions (CRT/RT, observation alone) after endoscopic treatment in patients with different depths of infiltration.

Positive lymphovascular invasion (LVI) was significantly associated with an increased risk of recurrence (OR = 4.01, 95% CI: 1.48–10.84, *P* = 0.006). Subgroup analysis indicated that LVI-positive patients in the chemoradiotherapy group had a particularly elevated recurrence risk (OR = 6.58, 95% CI: 1.06–40.77, *P* = 0.04). In contrast, no significant associations were observed in either the surgery group or the observation group (**Figure 4**). No significant relationship was found between tumor invasion depth and recurrence risk (OR = 1.26, 95% CI: 0.68–2.31, *P* = 0.46) (**Figure 5**).

#### 3.2.3 Publication bias

**Figure 6:**
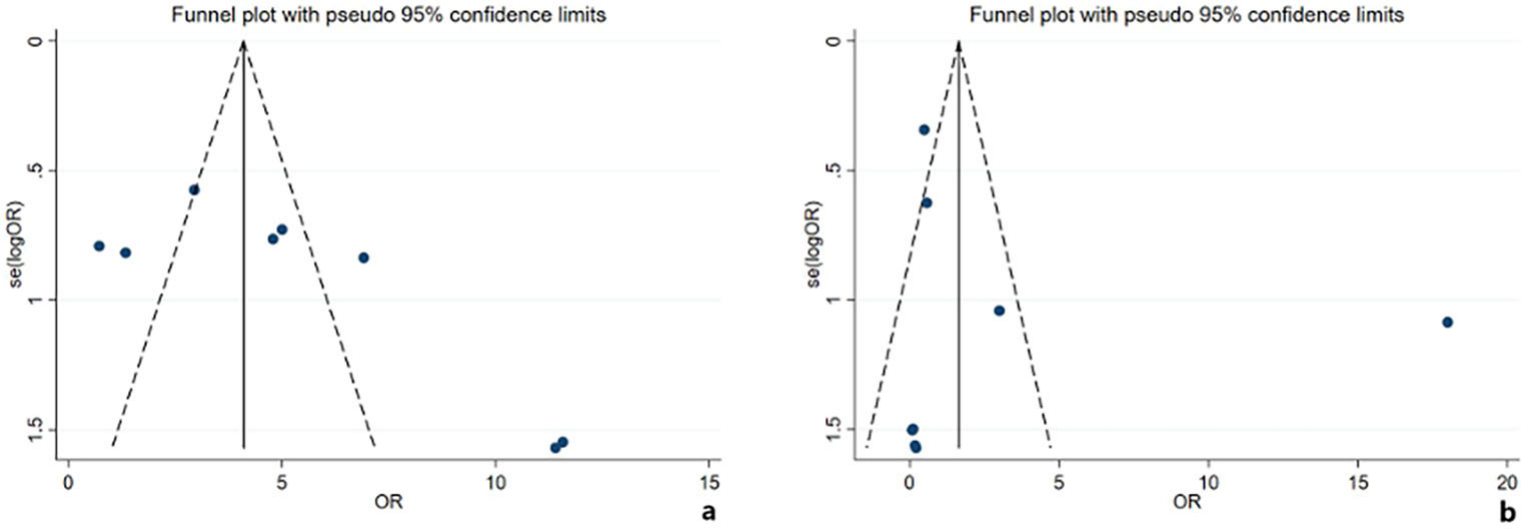
Funnel plots for the meta-analysis comparisons. **(a)** For observation *vs*. adjuvant treatment, the asymmetry suggests potential publication bias. **(b)** For adjuvant surgery *vs*. chemoradiotherapy, the dispersion is attributable to heterogeneity, with no statistical evidence of publication bias.

Assessment of publication bias in the comparison of observation versus adjuvant treatment involved visual inspection of the funnel plot, which indicated asymmetry (**Figure 6**), and Egger’s regression test, which showed a borderline significant result (*t* = 2.38, *P* = 0.055). This was corroborated by the trim-and-fill method, which estimated one missing study. After imputation, the pooled effect size retained statistical significance, indicating that the primary conclusion was robust. In contrast, for the comparison between adjuvant surgery and CRT, the observed funnel plot asymmetry (**Figure 6**) was not supported by statistical tests; Egger’s test was non-significant (*t* = 0.78, *P* = 0.467), and the trim-and-fill method estimated zero missing studies. This suggests that the dispersion was more likely attributable to substantial heterogeneity between studies than to publication bias.

#### 3.2.4 Sensitivity analysis

**Figure 7:**
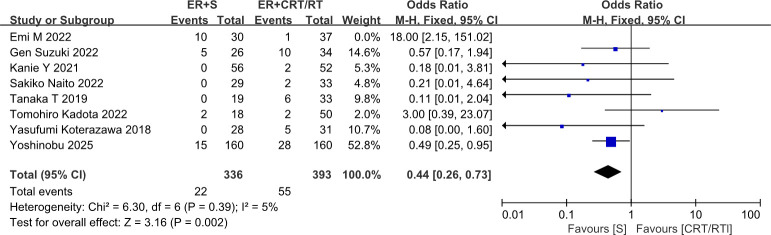
Forest plot comparing the recurrence rates after endoscopic resection followed by surgery versus endoscopic resection followed by chemoradiotherapy or radiotherapy, after excluding cases from Emi (17).

After exclusion of the Emi M 2022 study, heterogeneity was significantly reduced. The direction of effect remained consistent before and after exclusion; furthermore, after exclusion, the result became statistically significant, indicating that surgery was associated with a significantly lower recurrence risk compared to CRT/RT (OR = 0.44, 95% CI: 0.26–0.73, *P* = 0.002) (**Figure 7**). This finding suggested that the overall conclusion of “no difference” is not robust and is highly sensitive to the inclusion of this particular study. The Emi M 2022 study has a small sample size and an unusually high event rate, which likely contributed substantially to the observed heterogeneity. Therefore, future studies with larger sample sizes and standardized intervention protocols are recommended to further validate differences in treatment efficacy.

## 4 Discussion

Although ER is widely recognized as the first-line treatment for superficial esophageal cancer, the optimal management for patients after non-curative resection remains a critical issue. Our results indicate that lymphovascular invasion is a significant risk factor for recurrence in patients undergoing adjuvant treatment. Compared with observation alone, adjuvant treatment is associated with a lower recurrence rate; however, there is no significant difference in recurrence rates between adjuvant surgery and adjuvant CRT.

Based on the available evidence, adjuvant treatment is recommended for patients after non-curative ER, which aligns with the findings of Margarida et al. (24). In contrast to the findings of Jun Li et al., which suggest the superiority of adjuvant surgery (25, 26), our meta-analysis identifies no significant difference in recurrence rates between adjuvant surgery and adjuvant CRT. Moreover, sensitivity analyses reveal that this result is not robust, which collectively indicates that the evidence remains inconclusive. This uncertainty is largely attributable to substantial clinical heterogeneity, particularly in CRT protocols, which vary widely in chemotherapy regimens, radiotherapy doses, and treatment duration (**Supplementary Table S1**). Unlike the relatively standardized surgical approaches, the diversity of CRT regimens means that our pooled result averages potentially distinct therapeutic effects, thus precluding a direct comparison with studies that employ more homogeneous interventions. Owing to the limited number of studies, we cannot reliably quantify the influence of these variations. Therefore, the divergence from previous conclusions likely stems from these methodological variations rather than from a true absence of a differential effect. A retrospective study by Nakajo et al. proposes that for elderly patients with esophageal squamous carcinoma at high risk of lymph node metastasis after non-curative ESD, the Charlson Comorbidity Index (CCI) can guide the need for adjuvant treatment. For elderly patients aged 75 years or older with a CCI ≥2, observation after non-curative ESD is a suitable option, as they have a higher risk of non-cancer-related mortality (26). Moreover, the long-term survival rate for early-stage esophageal cancer is improving, so the patient’s quality of life is also a crucial consideration. In this context, CRT may be an effective alternative to surgery, as it is associated with fewer complications and better organ preservation. Therefore, for patients with non-curative esophageal cancer, the choice between adjuvant treatment options should be based on a comprehensive assessment of the patient’s general condition, comorbidities, and the risk–benefit ratio of each treatment.

Based on previous retrospective studies, the risk factors for recurrence after non-curative ER in superficial esophageal cancer include submucosal invasion, lymphovascular invasion, tumor diameter, positive margins, and multiple lesions (27). This analysis suggests that although tumor invasion depth does not reach statistical significance as an independent risk factor, a trend toward higher recurrence risk is observed. The discrepancy with prior findings may stem from several methodological limitations commonly encountered in such meta-analyses. These typically include an insufficient total sample size, which reduces statistical power (as evidenced by wide confidence intervals), significant heterogeneity in follow-up duration, and inadequate control for confounding factors. A critical and recurrent source of heterogeneity arises from the inconsistent definition and reporting of submucosal invasion depths: while a majority of studies define SM2 invasion as submucosal infiltration exceeding 200 μm, a minority fail to specify this critical boundary. This lack of a universally applied standard means that “SM1” or “SM2” cohorts are often not homogeneous across studies, an inconsistency that likely dilutes the true effect size and contributes to a non-significant pooled result. Despite the lack of statistical significance, the observed risk trend warrants clinical attention, and future multi-center studies with larger sample sizes and standardized protocols for both follow-up and histopathological reporting are needed for definitive validation.

The limitations of this study include the relatively small total sample size; the retrospective nature of most included studies, which may introduce selection and information bias; the small sample size of some individual studies; substantial clinical heterogeneity, particularly in the specific regimens of adjuvant chemoradiotherapy; and heterogeneity in patient baseline characteristics and follow-up duration across the studies.

In summary, lymphovascular invasion is a significant risk factor for recurrence in patients receiving adjuvant treatment after ER. Adjuvant treatment following non-curative ER for superficial esophageal cancer is beneficial, and in terms of recurrence rates, adjuvant surgery shows no significant superiority over adjuvant chemoradiotherapy. This study has implications for clinical practice; however, the included studies and cases are limited in number and predominantly retrospective, and the conclusions need to be validated by large-scale, prospective, multi-center studies.

## Data Availability

The datasets presented in this study can be found in online repositories. The names of the repository/repositories and accession number(s) can be found in the article/**Supplementary Material**.

## Supplementary material

Supplementary Table 1 Adjuvant treatment characterization.
