# Supplementary material for: Comparison of the efficacy of different treatments after non-curative endoscopic resection of superficial esophageal carcinoma: a meta-analysis
Source: Front Oncol. 2025 Dec 18;15:1709350. doi: 10.3389/fonc.2025.1709350 (PMC12756106; doi:10.3389/fonc.2025.1709350)
Supplement: Supplementary Table 1 — Adjuvant treatment characterization. [file Table1.docx]

**Supplementary Table 1.** Adjuvant treatment characterization

| Study | CT |  |  | RT | Surgery |
| --- | --- | --- | --- | --- | --- |
|  | Cis-platin | 5-Fluorouracil | Other | Dose (Gy) |  |
| Tomohiro Kadota 2022(Kadota et al., 2022) | 70 mg/m2/day | 700 mg/m2/day | nedaplatin (80 mg/m2/day,) and fluorouracil (800 mg/m2/day) | 41.4 | esophagectomy with three-field lymph node dissection |
| Yasufumi Koterazawa 2018(Koterazawa et al., 2018) | 70 mg/m2/day | 700 mg/m2/day |  | 41.4/50.4(negative resection margins/positive resection margins) | esophagectomy with lymph node dissection |
| Byeong Geun Song 2021(Song et al., 2021) |  |  |  |  | NR |
| Gen Suzuki 2022(Suzuki et al., 2022) | 75 mg/m2/day | 1000 mg/m2/day | used nedaplatin instead of cisplatin | 40/50(negative resection margins/positive resection margins) |  |
| Tanaka T 2019(Tanaka et al., 2019) | 100 mg/m2/day  80mg/m2/day  70mg/m2/day | 1000 mg/m2/day  800 mg/m2/day  700mg/m2/day | 5-Fluorouracil 200 mg/m2/day, cis-platin 4 mg/m2/day, and low-dose docetaxel (10 mg/m2(patients with frailty). | 51.4/50/45/41.4/40(n=1/1/8/21/2) | esophagectomy with 3-field lymph node dissection/Esophagectomy with 2-field lymph node dissection/Lower esophagectomy and proximal gastrectomy(n=7/11/1) |
| Hongna Lu 2024(Lu et al., 2024) |  |  |  | 50.4 |  |
| Emi M 2022(Emi et al., 2022) | 70 mg/m2/day | 700 mg/m2/day | used nedaplatin instead of cisplatin | 60 | subtotal esophagectomy |
| Kanie Y 2021(Kanie et al., 2021) | 70 mg/m2/day | 700 mg/m2/day |  | 41.4/50.4(negative resection margins/positive resection margins) | esophagectomy with two or three field lymph node dissection |
| Xu Yang 2023(Yang et al., 2023) |  |  |  | 41.4-60.0 |  |
| Sakiko Naito 2021(Naito et al., 2022) | 70 mg/m2/day | 700 mg/m2/day |  | 50.4 | esophagectomy with two or three field lymph node dissection |
| Osamu Hisano 2018(Hisano et al., 2018) |  |  |  | 61.4/50–50.4/40–41.4(n=1/7/5) |  |
| Mengxue Chen 2020(Chen et al., 2020) | NR |  |  | NR | NR |
| Yoshinobu 2025(Yamamoto et al., 2025) | 70-75 mg/m2(two courses) | 2800-4000 mg/m2(two courses) |  | 40-50.4 | esophagectomy with two or three field lymph node dissection |

The datasets generated during the current study are available in the Figshare repository, [DOI: 10.6084/m9.figshare.30812951].
